# Supplementary material for: A bridge-like lipid transfer protein is critical for generation of invasive stages in malaria parasites
Source: Nat Commun. 2026 Mar 28;17:3030. doi: 10.1038/s41467-026-70887-1 (PMC13036008; doi:10.1038/s41467-026-70887-1)
Supplement: Supplementary file 1 — Supplementary Information [file 41467_2026_70887_MOESM1_ESM.pdf]

A bridge-like lipid transfer protein is critical for generation of invasive stages in malaria parasites

Andrés Guillén-Samander[1], Nika Perepelkina[1], Vendula Horáčková[1], Hannah M. Behrens[1], Hely O. Rodriguez Cruz[2], Joëlle Paolo Mesén-Ramírez[1], Ana Ribeiro-Holbein[1], Per Haberkant[3], Frank Stein[3], Tobias Spielmann[1]

[1]Bernhard Nocht Institute for Tropical Medicine, Hamburg, Germany
[2]Department of Cell Biology, Yale University School of Medicine, Yale University, New Haven, CT, USA
[3]EMBL Proteomics Core, Heidelberg, Germany

Correspondence: Tobias Spielmann, spielmann@bnitm.de

Supplementary Information

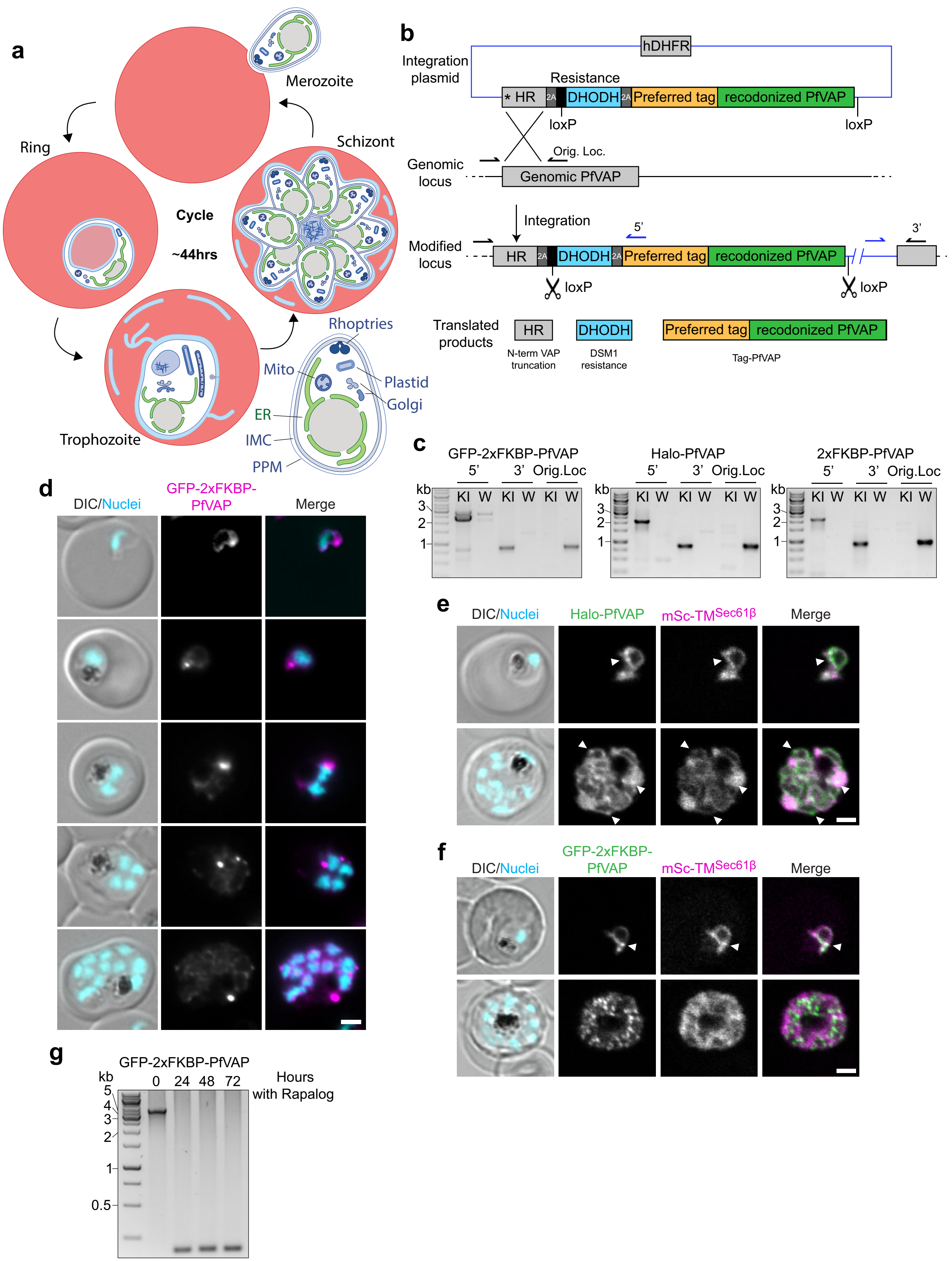

Supplementary Figure 1. PfVAP is an essential ER protein. a, Schematic of *P. falciparum* growth and stage development in RBCs. Key organelles for this study are highlighted in the merozoite zoom-in in the bottom right corner. **b**, Schematic depicting selection-linked integration (SLI) strategy to edit the

genomic locus of PfVAP to express N-terminally tagged versions of the protein under its original promoter. The expression of the drug resistance gene yDHODH fused to PfVAP with a skip peptide (T2A) was used to select edited parasites. The asterisk indicates a stop codon. **c**, Agarose gels showing PCR products amplified from genomic DNA of the indicated cell lines confirming correct integration of genome-modified parasites: 5' and 3' are amplicons generated across the integration junctions and the original locus amplicon (Orig. Loc.) shows presence or absence of the unmodified locus, comparing parental (W) and knock-in (KI) lines. **d**, Fluorescence microscopy images of GFP-2xFKBP-$PfVAP^{endo}$, showing the artifactual formation of PfVAP accumulations in the ER across all parasite stages, potentially mediated by GFP dimerization. **e-f**, Representative confocal images of parasites with endogenously edited PfVAP and episomally co-expressing $mSc\text{-}TM^{Sec61\beta}$, reflecting an enrichment at hotspots in the ER (arrowheads). This is seen with Halo-tagged PfVAP (d), and artifactually exacerbated with GFP-tagged PfVAP (e). **g**, Agarose gels with PCR products amplified from genomic DNA confirming the excision of PfVAP upon rapalog-mediated diCre activation.
DIC, differential interference contrast; Nuclei, Hoechst 33342; scale bars, 2 µm.

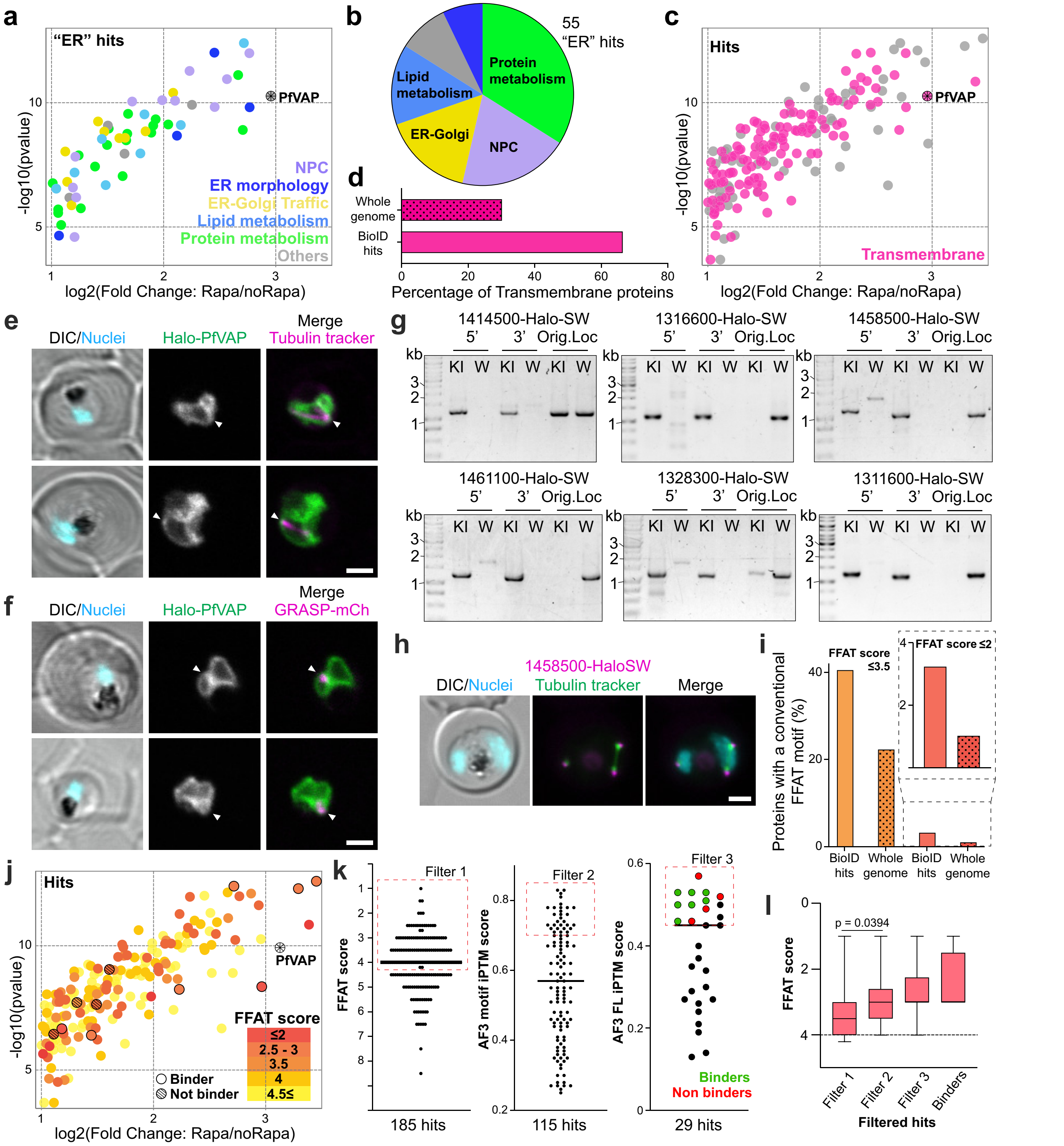

Supplementary Figure 2. PfVAP DiQ-BioID hits are enriched in ER and FFAT-motif containing proteins. a, PfVAPDiQ-BioID hits annotated as "ER" proteins in Fig. 2c classified by function. NPC: Nuclear Pore Complex. **b**, Pie chart showing proportion of proteins from (a) with the indicated functional classification. **c**, PfVAP DiQ-BioID hits from Fig. 2c annotated by the presence of a transmembrane domain. **d**, Graph comparing the percentage of transmembrane proteins in the hits in (c) with the proportion of all proteins annotated from the *P. falciparum* genome. **e-f**, Representative confocal images showing Halo-PfVAP and the Centriolar Plaque (e) or the Golgi (f), as labelled by tubulin tracker and episomally expressed GRASP-mCherry (GRASP-mCh), respectively. Arrowheads show areas with overlap. Both compartments are annotated as "Others" in Fig. 2c and are in proximity

to PfVAP. **g**, Agarose gels showing PCR products amplified from genomic DNA of the indicated cell lines confirming correct integration of genome-modified parasites. Features as in Supplementary Fig. 1c. In the case of the PF3D7_1414500-Halo-SW line, there is still a significant population of unedited parasites, with no fluorescence signal, but this does not affect localization experiments which was the sole purpose of this line for this study. **h**, Representative fluorescence microscopy images showing PF3D7_1458500 (SAS4) adjacent to the tubulin foci that labels the inner centriolar plaque, confirming the localization of this candidate to the outer centriolar plaque. **i**, PfVAP DiQ-BioID hits from Fig. 2c colored by the score of their highest scoring FFAT-motif, calculated based on the opisthokont FFAT consensus motif [1]. The proteins containing the motifs analyzed in Fig. 3b-c are highlighted with a black stroke, of which those determined as non-binders are filled with diagonal lines. **j**, Bar chart showing percentages of proteins containing high-scoring FFAT motifs of the 185 PfVAP DiQ-BioID hits (BioID hits) and in all proteins encoded in the *P. falciparum* genome (whole genome) as calculated in Slee et al [1]) using a cut-off of 3.5 or lower, or 2 or lower (inlay). **k**, Graphs showing the distribution of FFAT and AF3 iPTM scores across the PfVAP DiQ BioID hits and the different filtered groups. The filters were FFAT score <4, AF3 motif iPTM score >0.7 and AF3 FL iPTM score >0.45. **l**, Box and whiskers plot showing FFAT score distribution across filtered hits. P-value, unpaired t-test.
DIC, differential interference contrast; Nuclei, Hoechst 33342; scale bars, 2 µm.

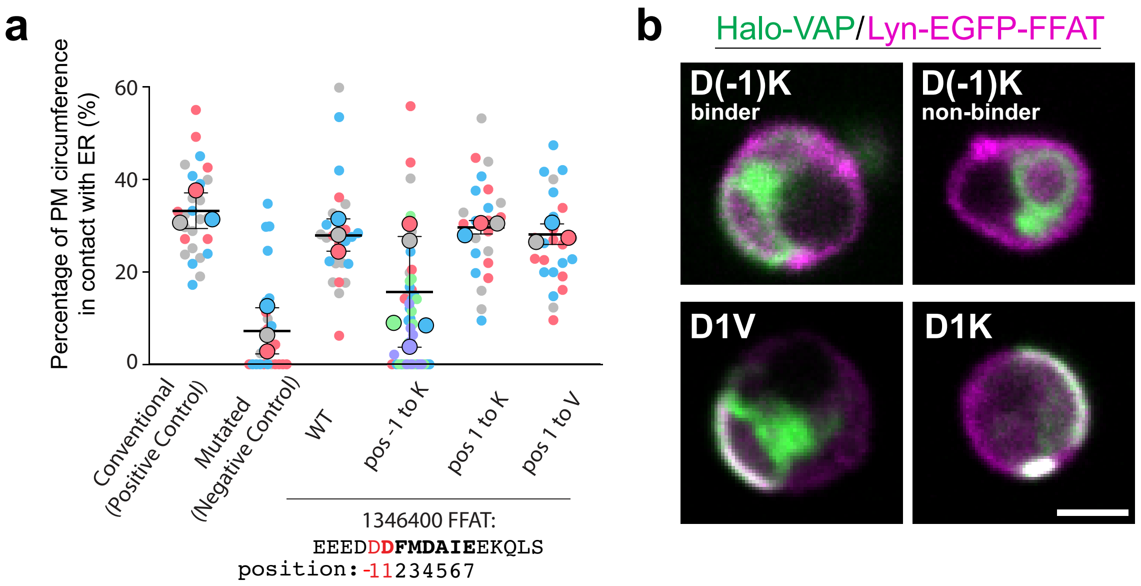

Supplementary Figure 3. Binding of the FFAT motif of PfVPS13L1 (PF3D7_1346400) to PfVAP after single point mutations. a, Quantification of ER-PM MCSs distance (percentage of total PM circumference), shown as superplot[2], for Lyn-FFAT motif constructs (assay explained in Fig. 1e) using point mutants of the motif found in PfVPS13L1. Positive and negative control data are the same as in Fig. 1g and PfVPS13L1 data is the same as in Fig. 2g, included in this graph for reference. For motifs with mutations D(-1)K, D1K and D1V, data was taken from 5, 3 and 3 independent experiments with a total of 47, 23 and 24 parasites (1- or 2-nuclei trophozoite stage), respectively; colors indicate independent experiments (small dots, individual parasites; large dots, average of each experiment; black lines, mean and SD). More replicates were carried out for the motif with mutation D(-1)K due to inconsistencies in its binding to PfVAP, probably indicating a reduced affinity. **b**, Representative confocal microscopy example images of Halo-PfVAP^{endo} parasites episomally expressing the PfVPS13L1 FFAT motif with the indicated point mutations and used to quantify the resulting ER-PM MCSs distance. An example of binding and no-binding is shown for the motif with the D(-1)K mutation. Scale bar, 2 µm.

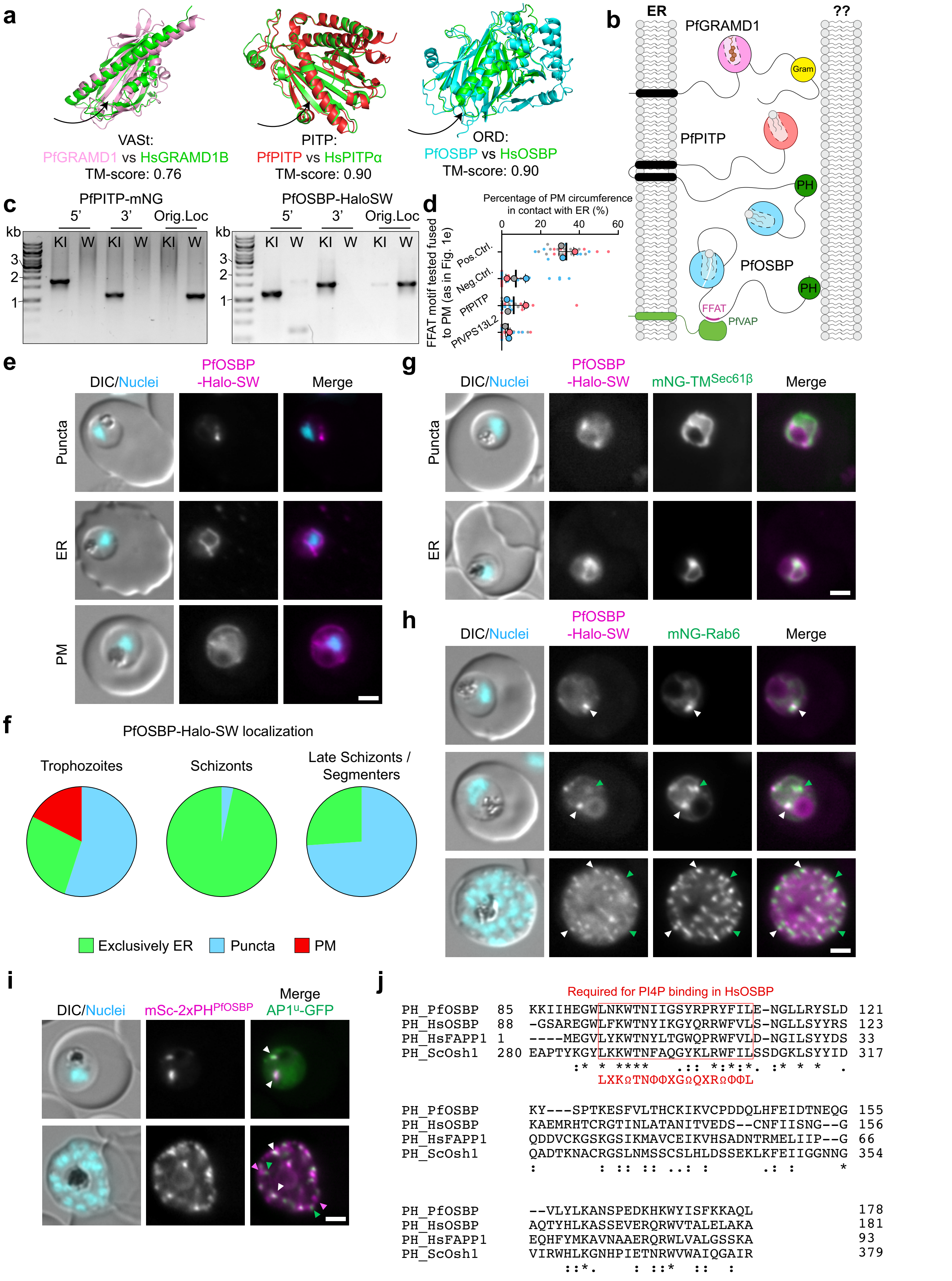

Supplementary Figure 4. The Shuttle-like LTP PfOSBP mediates ER-Golgi MCSs. a, Structural

alignment using TM-align[3] between the AlphaFold predicted structures of domains found in *P. falciparum* LTPs and structures of human LTPs: ORD of HsOSBP (PDB 7V62[4]), HsPITPα (PDB 1UW5[5]) and VASt of HsGRAMD1B (AF-Q3KR37-F1-v4[6,7]). **b**, Schematics indicating potential arrangement of the shuttles found in this study at ER-MCSs. **c**, Agarose gels showing PCR products amplified from genomic DNA of the indicated cell lines confirming correct integration of genome-modified parasites. Features as in Supplementary Fig. 1c. **d**, Quantification of ER-PM MCSs distance (percentage of total PM circumference), shown as superplot[2], for Lyn-FFAT motif constructs (assay explained in Fig. 1e) using motifs found in PfPITP (motif centered around Y167: DYNESED) and PfVPS13L2 (motif centered around Y603: DYFTTKE). Positive and negative control data are same as in Fig. 1g and included for reference. For PfPITP and PfVPS13L2 motifs data was taken from n=3 independent experiments with a total of 19 and 25 parasites (1- or 2-nuclei trophozoite stage), respectively; colors indicate independent experiments (small dots, individual parasites; large dots, average of each experiment; black lines, mean and SD). **e**, Representative fluorescence microscopy images of PfOSBP-Halo-SW dynamic localization to the ER (expected due to the interaction of its FFAT with PfVAP), either exclusively to the ER (ER) or with puncta or with the PM (PM). **f**, Proportion of the phenotypes from (e) from a total of 40 trophozoites, 29 schizonts and 23 late schizonts/segmenters from 4 independent experiments. **g-h**, Representative fluorescence microscopy images showing PfOSBP-Halo-SW edited parasites with episomal expression of the ER marker mNG-TM$^{Sec61\beta}$ (g) or the Golgi marker mNG-Rab6 (h) in trophozoites. The PfOSBP-Halo-SW puncta colocalize with both markers, indicating that these are hotspots where the two organelles overlap. Arrowheads in (h) indicate colocalization (white) or only Rab6 positive (green) puncta. **i**, The PH domain of PfOSBP is recruited to the Golgi in trophozoites and partially in schizonts, as seen by colocalization of an episomal construct encoding a mScarlet tagged tandem of two copies of this PH domain (mSc-2xPH^{PfOSBP}) with the μ subunit of the AP-1 complex expressed from the endogenous locus [AP-1μ-GFP (endo)]. Arrowheads in schizonts indicate colocalization (white), only mSc-2xPH^{PfOSBP} (magenta), or only AP-1μ (green) puncta. **j**, Sequence alignment between PH domain of PfOSBP and human and yeast homologues expected to bind PI4P. The red box indicates the PI4P-binding wedge conserved in PfOSBP. Ω, Φ and X, indicate aromatic, hydrophobic and any residue, respectively. In the alignment, **.** , **:** and *****, indicate similar, highly similar, and identical residues, respectively. DIC, differential interference contrast; Nuclei, Hoechst 33342; scale bars, 2 μm.

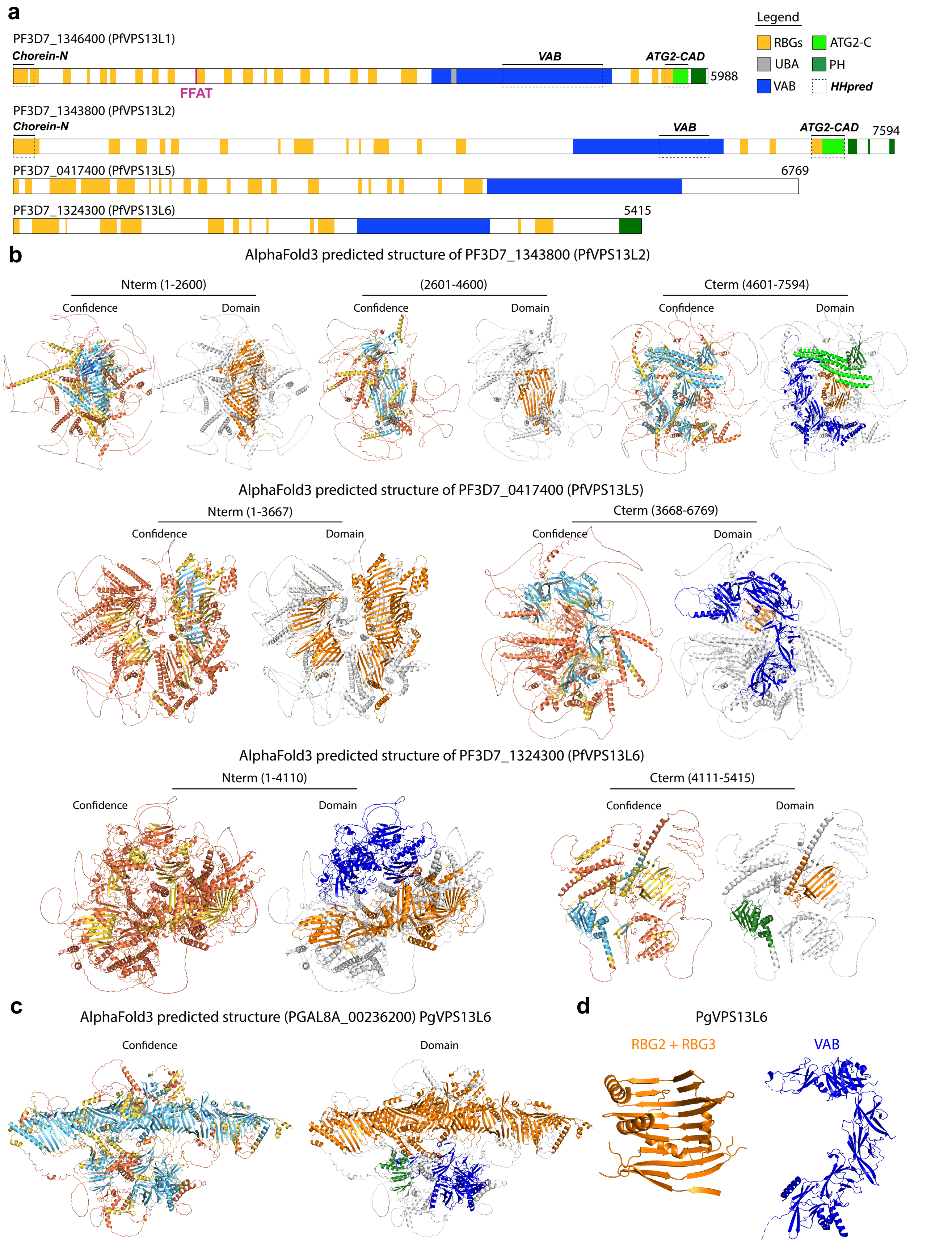

Supplementary Figure 5. AlphaFold3 predicted structures of *P. falciparum* VPS13L proteins identified in the PfVAP DiQ-BioID. a, Domain cartoons of the four VPS13L proteins identified in the PfVAP DiQ-BioID. The dashed boxes indicate domains that were identified by HHpred. **b**, Confidence-

and domain-colored predicted structures of PfVPS13L proteins. PfVPS13L1 structure is detailed in Supplementary Fig. 7. **c**, Confidence- and domain-colored predicted structure of the *P. gallinaceaum* homolog of PfVPS13L6 (PgVPS13L6). **d**, Examples of RBGs and VAB identified in PgVPS13L6.

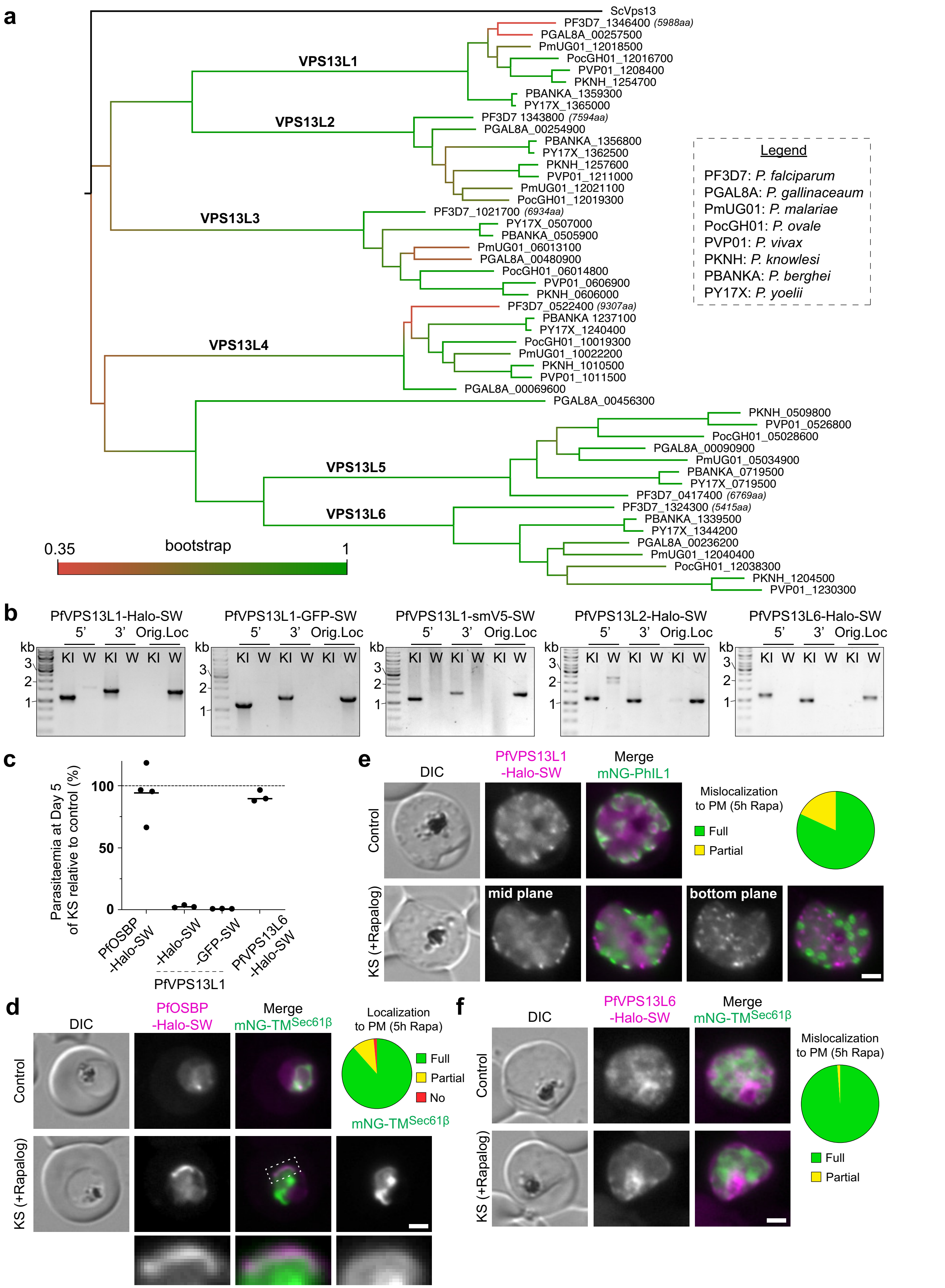

Supplementary Figure 6. PfVPS13L1 is an essential BLTP. a, Phylogenetic tree of the six VPS13-like proteins identified in *P. falciparum* and their homologues in other *Plasmodium* species. **b**, Agarose

gels showing PCR products amplified from genomic DNA of the indicated cell lines confirming correct integration of genome-modified parasites. Features as in Supplementary Fig. 1c. **c**, Graph summarizing growth defects observed in independent replicates (each indicated by a dot; bar, mean) after 5 days upon knock-sideways (KS) of the indicated protein using the parasite lines generated in (Supplementary Fig. 4c and 6b) episomally expressing the Lyn-FRB mislocalizer. P-values, paired t test between the KS and control: PfOSBP-Halo-SW^{endo}, 0.2971; PfVPS13L1-Halo-SW^{endo}, <0.0001; PfVPS13L1-GFP-SW^{endo}, <0.0001; PfVPS13L6-Halo-SW^{endo}, 0.0832. **d-f**, Representative fluorescence microscopy images and quantification of the KS of the indicated target proteins. Mislocalization of PfOSBP to the PM (d) also resulted in the formation of ER-PM MCSs, as seen by the ER marker (also shown in the 3.5x enlargement of the boxed area), confirming its attachment to the ER via PfVAP. Similarly, mislocalization of PfVPS13L1-Halo-SW resulted in puncta at the PM (e). Pie charts show localization after rapalog addition, (total of 71 (d), 48 (e) and 56 (f) parasites across 2 experiments for each parasite line).

DIC, differential interference contrast; Nuclei, Hoechst 33342; scale bars, 2 µm.

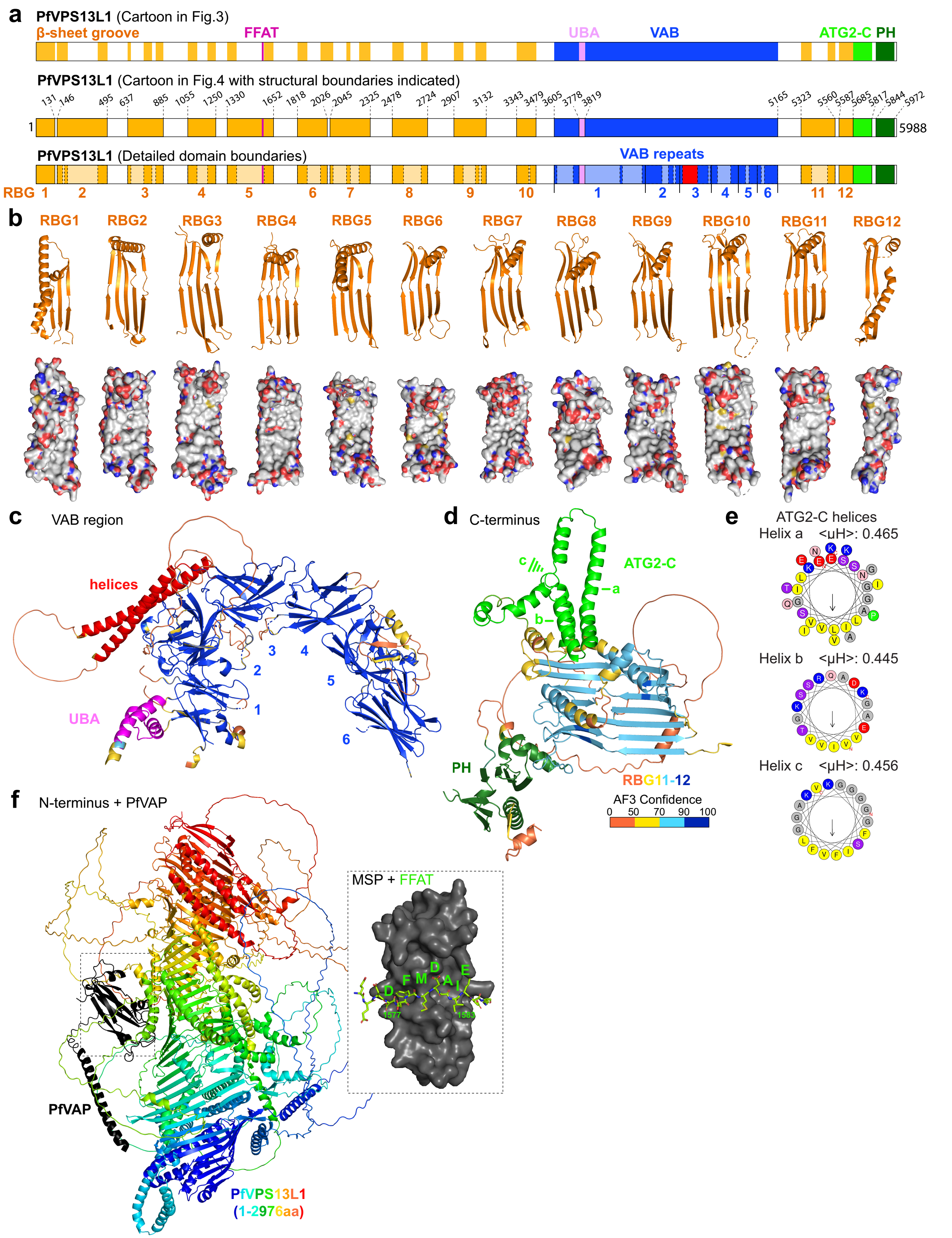

Supplementary Figure 7. Structural details of PfVPS13L1. a, PfVPS13L1 domain cartoons with structural details annotated. Top, annotation of all the pieces that form the lipid transferring β-sheet rod, similarly to the cartoons in Fig. 4d. In the mid cartoon, these pieces are grouped to indicate the multiple repeating units (RBG domains) forming the rod, and the boundaries of each annotated region

are indicated. Bottom, indicates the disordered regions that are part of each domain in a lighter shade of color. **b-d**, Details of AlphaFold3 predicted PfVPS13L1 structure. PfVPS13L1 is composed of: 12 RBG repeats (b), which have a hydrophobic face to accommodate the hydrophobic tails of lipids; a VPS13 Adapter Binding (VAB) domain formed by 6 β-strand repeats (c) that sticks out of the RBG-formed rod between RBG10 and RBG11; a UBA domain that is attached to the first repeat in the VAB domain (c); and a tandem of ATG2-C and PH domains found at the C-terminal end of the rod (d). A pair of helices stick out of the third repeat of the VAB domain, a feature not observed in any other eukaryotic VPS13 (c). The surface representation in (b) is colored by element (Oxygens are red, Nitrogens are blue and Carbons white), the ribbon representations in (c) and (d) are colored by AlphaFold3 confidence with the indicated domains colored according to the annotated cartoon in (a). **e**, Heliquest[8] predictions of the hydrophobic moment of three helices of the ATG2-C domain confirming their amphipathicity, which allows the C-terminal end of the rod to closely interact with lipid bilayers. **f**, AlphaFold3 prediction of the interaction between the N-terminal region (residues 1-2976) of PfVPS13L1 (colored by N- to C-terminus) and PfVAP. The MSP domain of PfVAP is predicted to bind the FFAT motif tested in Fig. 3b-c (inset), which would anchor the N-terminal end of the rod to the ER.

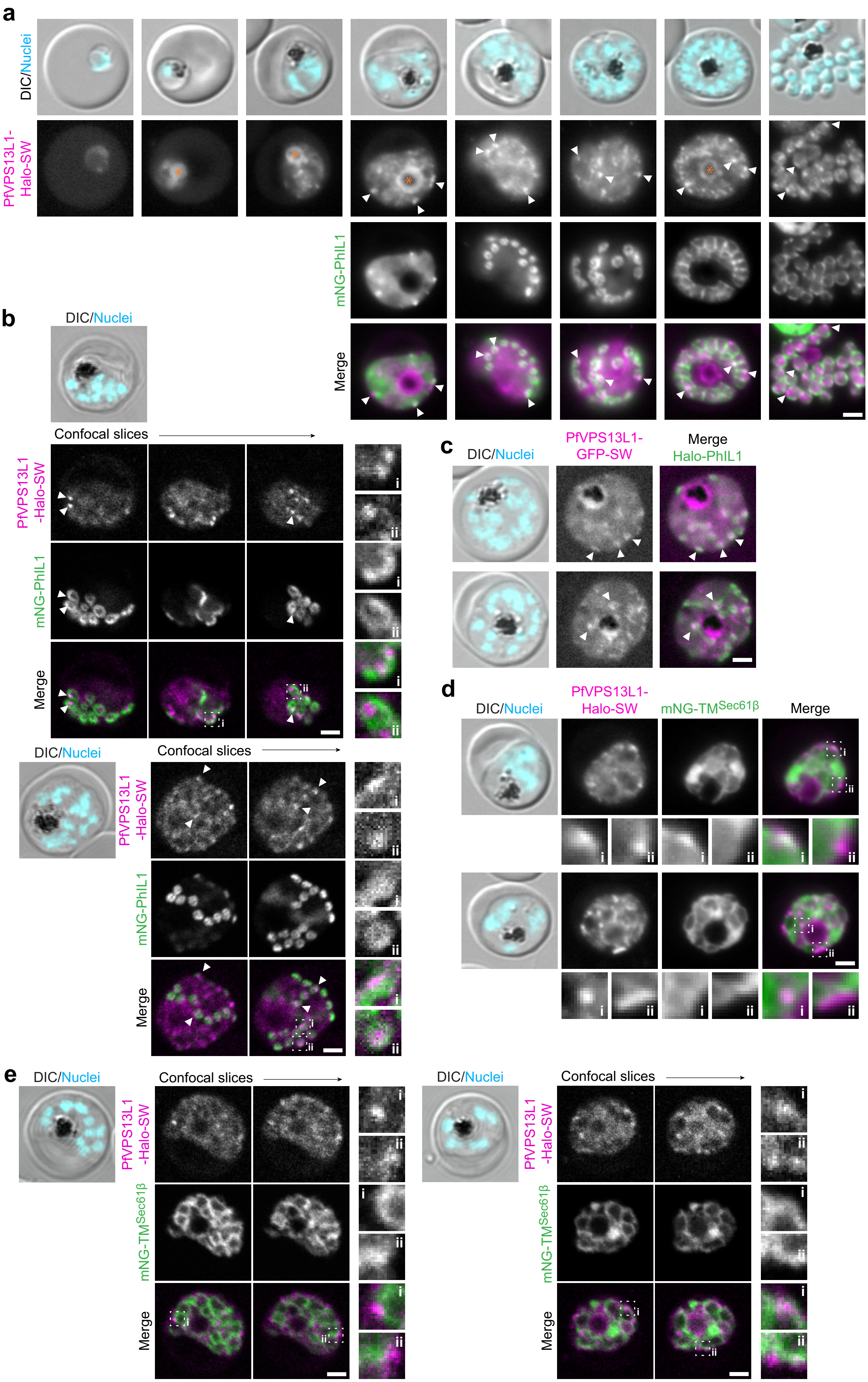

a
DIC/Nuclei
PfVPS13L1-Halo-SW
mNG-PhIL1
Merge
b
DIC/Nuclei
Confocal slices
PfVPS13L1-Halo-SW
mNG-PhIL1
Merge
c
DIC/Nuclei
PfVPS13L1-GFP-SW
Merge
Halo-PhIL1
d
DIC/Nuclei
PfVPS13L1-Halo-SW
mNG-TM^{Sec61β}
Merge
e
DIC/Nuclei
Confocal slices
PfVPS13L1-Halo-SW
mNG-TM^{Sec61β}
Merge

Supplementary Figure 8. PfVPS13L1 colocalizes with the IMC during its early stages of formation. a, Representative fluorescence microscopy images of parasites expressing PfVPS13L1-Halo-SW from the endogenous locus (Supplementary Fig. 6b) and episomally expressing mNeon tagged PhIL1 (mNG-PhIL1) as an IMC marker. Little to no expression is observed in rings and trophozoites (orange asterisk indicates food vacuole background typical for high exposure images), whereas foci that colocalize with the IMC marker are observed in schizont stages (arrowheads). The protein remains in foci even after completion of IMC formation. **b**, Representative confocal microscopy images [using the cell line in (a)] showing PfVPS13L1 foci localize to a subsection of the IMC or directly adjacent to it. **c**, Representative fluorescence microscopy images of parasites expressing PfVPS13L1-GFP-SW from the endogenous locus (Supplementary Fig. 6b), which is in foci that colocalize with the episomally expressed IMC marker Halo-PhIL1 in early stages of IMC formation (arrowheads). **d-e**, Endogenous PfVPS13L1-Halo-SW hotspots are also colocalizing with or directly adjacent to the ER marker mNG-TM$^{Sec61\beta}$, as seen by fluorescence (d) or confocal (e) microscopy. 3x enlargements of two boxed regions (i and ii in the merged slices) are shown to the right or at the bottom of each panel in (b-e).

DIC, differential interference contrast; Nuclei, Hoechst 33342; scale bars, 2 µm.

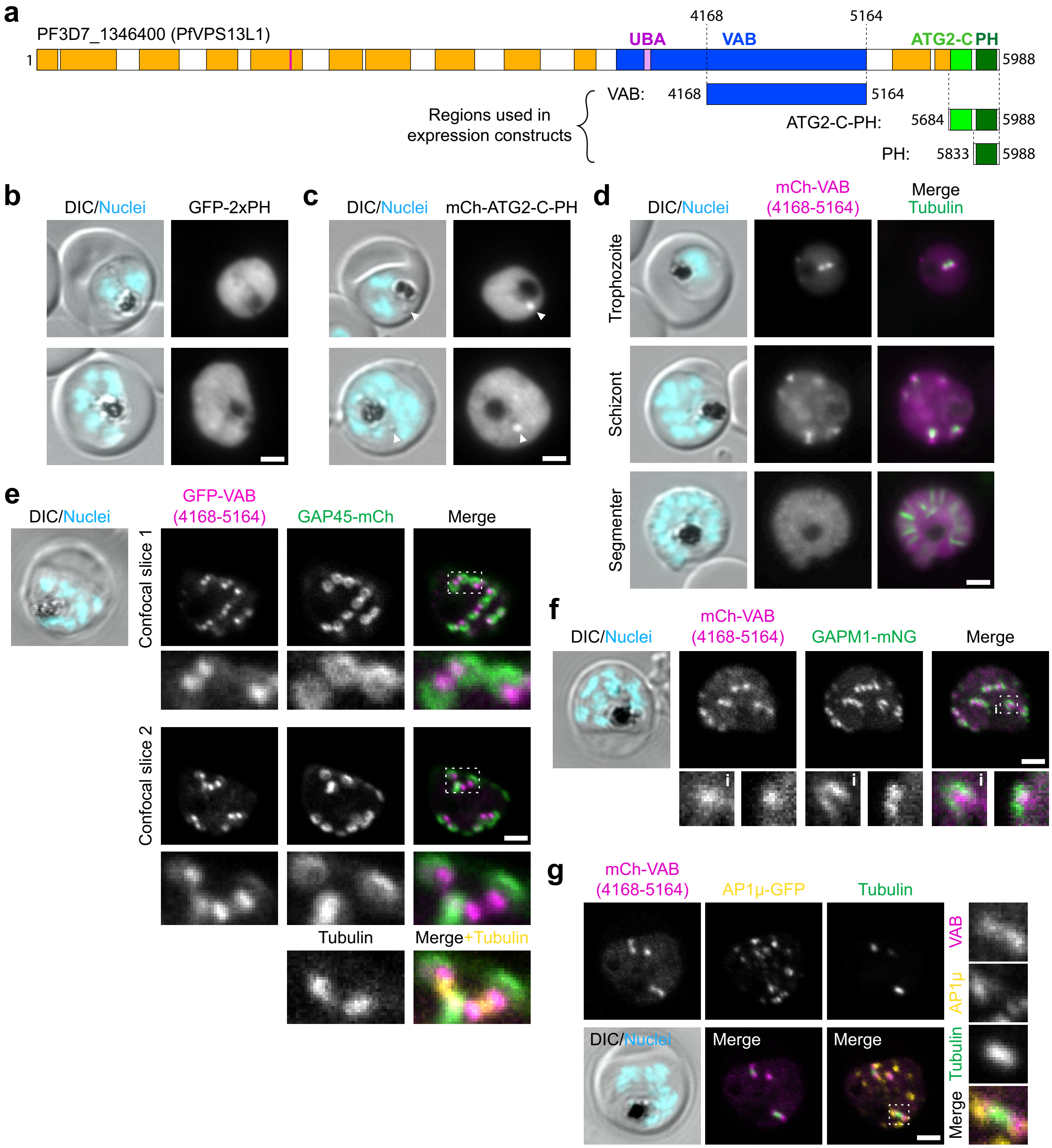

Supplementary Figure 9. The C-terminal VAB domain of PfVPS13L1 regulates its localization to the IMC. a, PfVPS13L1, full length and truncations, domain cartoons. **b-d**, Representative fluorescence microscopy images showing the localization of episomally expressed C-terminal domains of PfVPS13L1 indicated in (a). The parasites expressing the VAB construct were stained with tubulin tracker (d). **e-f**, Confocal images of episomally expressed GFP- (e) or mCh- (f) tagged VAB showing partial colocalization with or a localization adjacent to the IMC markers GAP45-mCh (e) or GAPM1-mNG (f). The VAB hotspot is also adjacent to the tubulin tracker-stained inner centriolar plaque. **g**, Confocal images of tubulin tracker-stained parasites episomally expressing mCh-VAB and the Golgi apparatus as seen by AP-1µ-GFP (endo). Images in (g) are a maximum intensity Z-projection of two confocal slices. 3x enlargements of boxed regions in the merged slices are shown at the bottom or to the right of each panel in (e-g). The second enlargement in (f) is taken from a different confocal slice. DIC, differential interference contrast; Nuclei, Hoechst 33342; scale bars, 2 µm.

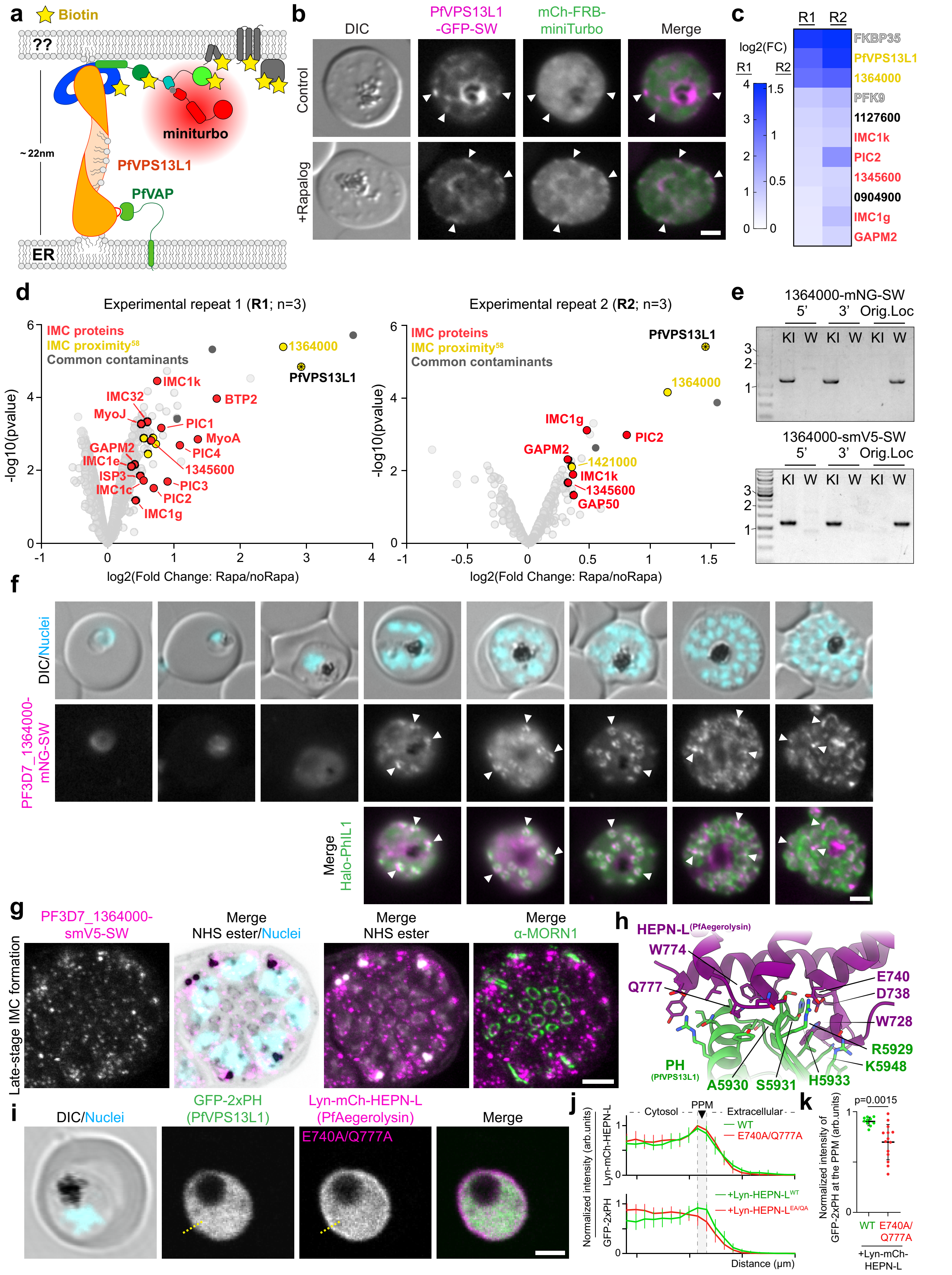

Supplementary Figure 10. The C-terminal end of the PfVPS13L1 bridge interacts with the IMC. a, Schematic of PfVPS13L1 DiQ-BioID experiment. The labeling radius of the miniTurbo biotinylizer is

smaller than the length of the PfVPS13L1 bridge (~10 nm[9]), which is expected to allow for selective labelling of proteins in proximity to the C-terminal end of PfVPS13L1. **b**, Representative fluorescence microscopy images of parasites expressing PfVPS13L1-GFP-SW from the endogenous locus (Supplementary Fig. 6b) and episomally expressing the mCh-FRB-miniTurbo biotinilyzer, showing the rapalog-mediated recruitment of the biotinilyzer to the C-terminus of PfVPS13L1 as outlined in (a). Arrowheads indicate PfVPS13L1-GFP-SW foci. **c**, Heatmap with the 11 proteins found to pass the thresholds in (c) in both experimental repeats (R1 and R2) from (c). IMC proteins color coded as in d. FKBP35 and PFK9 are common BioID contaminants. **d**, Individual LC-MS/MS experiments (each with 3 independent replicates) of C-terminal PfVPS13L1 DiQ-BioID. The left panel is the same experiment as shown in Fig. 6a. Proteins colored as indicated when meeting the cut offs (enrichment of at least 25% with a p value of at least 0.07). Moderated t-test was applied as implemented in the limma package. **e**, Agarose gels showing PCR products amplified from genomic DNA of the indicated cell lines confirming correct integration of genome-modified parasites. Features as in Supplementary Fig. 1c. **f**, Representative fluorescence microscopy images of the parasite line with endogenously tagged PF3D7_1364000-mNG-SW (e) and episomally expressing the IMC marker Halo-PhIL1 along the intraerythrocytic cycle. Colocalization with a subsection of the IMC is observed since early stages of its formation, and the protein remains in foci even after completion of IMC formation, similar to what was observed for PfVPS13L1 (Supplementary Fig. 8a). Arrowheads indicate PF3D7_1364000 foci. **g**, Ultrastructure Expansion Microscopy (U-ExM) images of parasites in late-stages of IMC formation expressing PfAegerolysin-smV5-SW from the endogenous locus co-immunostained for the basal complex marker α-MORN1. **h**, Ribbon representation of the Alphafold3-predicted interaction between the HEPN-L domain of PfAegerolysin and the PH domain of PfVPS13L1 (iPTM=0.84). The interface residues are shown as sticks colored by element and the most conserved residues participating in the interaction are highlighted. **i**, Representative confocal images of parasites episomally expressing the PH domain of PfVPS13L1 (GFP-2xPH) with episomal co-expression of the HEPN-L domain of PfAegerolysin artificially localized to the PM with a Lyn-targeting sequence and with two point mutations (E740A/Q777A) in the surface of interaction with the PH domain. **j**, Intensity measurements across a traced line traversing the PM, as in Fig. 6h, of the Lyn-mCh-HEPN-L WT or mutant construct (top), and the GFP-2xPH domain construct when co-expressed with the indicated Lyn-mCh-HEPN-L constructs (bottom). The bottom graph is also included in Fig. 6h. The Lyn-mCh-HEPN-L intensity peak (shaded region) corresponds to the parasite PM (PPM). **k**, Average normalized intensity of GFP-2xPH at the PPM, as defined by the peak of Lyn-mCh-HEPN-L intensity. (black lines, mean with SD; p-value, unpaired t test).
DIC, differential interference contrast; Nuclei, Hoechst 33342; scale bars, 2 µm in (b, f & i) and 5 µm in (g).

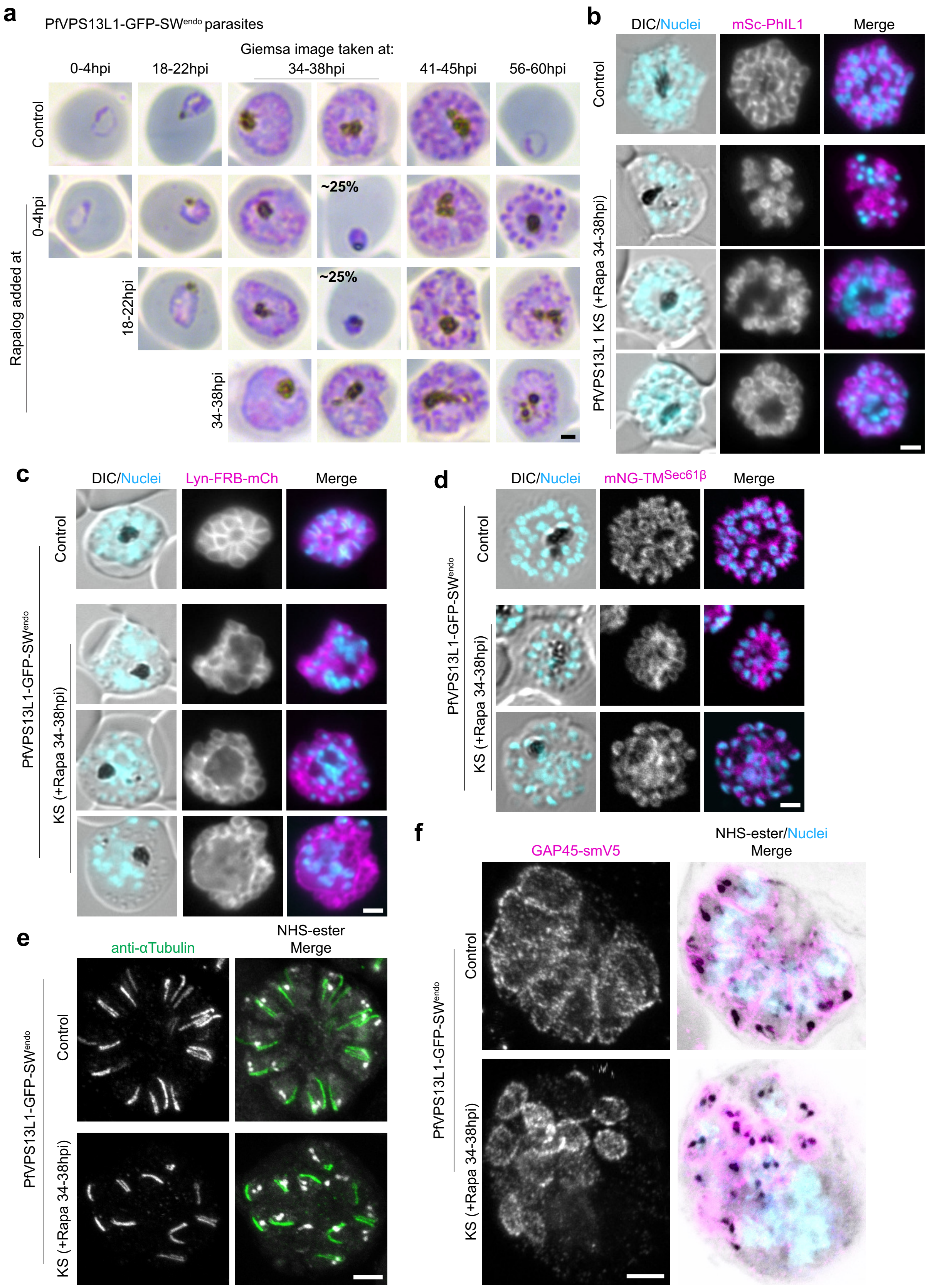

Supplementary Figure 11. PfVPS13L1 loss-of-function leads to severe segmentation defects. a, Representative example microscopy images of the Giemsa smears of PfVPS13L1-GFP-SW^{endo} control and KS parasites (induced at different time points through addition of rapalog) from the stage quantification experiment in Fig. 7a**. b-c**, Fluorescence microscopy images of control (same parasites without rapalog) and late (34-38hpi) induced PfVPS13L1 KS schizonts arrested before egress with

compound 2 and episomally expressing the IMC marker PhIL1 (b) or the PM marker Lyn-FRB-mCh (c). **d**, Representative confocal microscopy images of PfVPS13L1-Halo-SW^{endo} control and late (34-38hpi) induced KS compound 2-arrested schizonts episomally expressing the ER marker mNG-TM$^{Sec61\beta}$. **e-f**, U-ExM images of control and late-induced PfVPS13L1 KS compound 2-arrested schizonts immunostained with anti-αTubulin (e) or anti-V5 (in GAP45-smV5 expressing parasites; f). No striking differences were observed for tubulin, which appeared as extended structures in segmented schizonts (e), whereas IMC was smaller (f), as observed in Fig. 8f. U-ExM images are maximum intensity projections of Z-slices, with 4 (e, control), 33 (e, KS), 7 (f, control) and 6 (f, KS) slices.
DIC, differential interference contrast; Nuclei, Hoechst 33342; scale bars, 2 µm in (a-d) and 5 µm in (e & f).

Supplementary references:

1. Slee, J. A. & Levine, T. P. Systematic prediction of FFAT motifs across eukaryote proteomes identifies nucleolar and eisosome proteins with the predicted capacity to form bridges to the endoplasmic reticulum. *Contact (Thousand Oaks)* **2**, 1–21 (2019).
2. Lord, S. J., Velle, K. B., Mullins, R. D. & Fritz-Laylin, L. K. SuperPlots: Communicating reproducibility and variability in cell biology. *J. Cell Biol.* **219**, (2020).
3. Zhang, Y. & Skolnick, J. TM-align: a protein structure alignment algorithm based on the TM-score. *Nucleic Acids Res.* **33**, 2302–9 (2005).
4. Kobayashi, J. *et al.* Ligand Recognition by the Lipid Transfer Domain of Human OSBP Is Important for Enterovirus Replication. *ACS Infect. Dis.* **8**, 1161–1170 (2022).
5. Tilley, S. J. *et al.* Structure-function analysis of human [corrected] phosphatidylinositol transfer protein alpha bound to phosphatidylinositol. *Structure* **12**, 317–26 (2004).
6. Jumper, J. *et al.* Highly accurate protein structure prediction with AlphaFold. *Nature* **596**, 583–589 (2021).
7. Varadi, M. *et al.* AlphaFold Protein Structure Database in 2024: providing structure coverage for over 214 million protein sequences. *Nucleic Acids Res.* **52**, D368–D375 (2024).
8. Gautier, R., Douguet, D., Antonny, B. & Drin, G. HELIQUEST: a web server to screen sequences with specific alpha-helical properties. *Bioinformatics* **24**, 2101–2 (2008).
9. Kim, D. I. *et al.* Probing nuclear pore complex architecture with proximity-dependent biotinylation. *Proc. Natl. Acad. Sci. U. S. A.* **111**, E2453-61 (2014).
