# Supplementary material for: A bridge-like lipid transfer protein is critical for generation of invasive stages in malaria parasites
Source: Nat Commun. 2026 Mar 28;17:3030. doi: 10.1038/s41467-026-70887-1 (PMC13036008; doi:10.1038/s41467-026-70887-1)
Supplement: Supplementary file 3 — Description of Additional Supplementary Files [file 41467_2026_70887_MOESM3_ESM.pdf]

Description of Additional Supplementary Files

File Name: Supplementary Data 1
Description: PfVAP DiQ-BioID results.

File Name: Supplementary Data 2
Description: Highest scoring FFAT motifs in P. falciparum proteome and DiQ-BioID results.

File Name: Supplementary Data 3
Description: Filtering of PfVAP DiQ-BioID results to identify potential direct interactors.

File Name: Supplementary Data 4
Description: PfVPS13L1 C-terminus DiQ-BioID results.

File Name: Supplementary Data 5
Description: Oligos and DNA fragments synthesized for this study.

File Name: Supplementary Data 6
Description: Genomic sequences of loci edited in this study.

File Name: Supplementary Movie 1
Description: PfVPS13L1 structure. Rotating AlphaFold3 predicted structure (N-terminus to the left). Structure representations transition from 1) Ribbon representation colored by AlphaFold3 prediction confidence (as in Fig. 5b), 2) Ribbon representation colored by domain annotation (as in Fig. 5c), and 3) Surface representation where the lipid transfer groove was colored by element (Oxygen, red; Nitrogen, blue; Carbon, white; Sulfur, yellow).

File Name: Supplementary Movie 2
Description: PfVPS13L1 mislocalization leads to reduced IMC growth and faulty egress. Timelapse confocal microscopy of PfVPS13L1-GFP-SWendo parasites episomally expressing the IMC marker Halo-PhIL1 and the PM mislocalization construct Lyn-FRB-mCh without (control) or with rapalog added at 34-38hpi (knock-sideways). Zstacks were acquired every 20min, starting at 36-40hpi. 4 cells for each condition are included. Scale bar, 2 µm.
