# Supplementary material for: A bridge-like lipid transfer protein is critical for generation of invasive stages in malaria parasites
Source: Nat Commun. 2026 Mar 28;17:3030. doi: 10.1038/s41467-026-70887-1 (PMC13036008; doi:10.1038/s41467-026-70887-1)
Supplement: Supplementary file 12 — Reporting Summary [file 41467_2026_70887_MOESM12_ESM.pdf]

nature portfolio

Corresponding author(s): Dr. Tobias Spielmann

Last updated by author(s): Feb 25, 2026

Reporting Summary

Nature Portfolio wishes to improve the reproducibility of the work that we publish. This form provides structure for consistency and transparency in reporting. For further information on Nature Portfolio policies, see our Editorial Policies and the Editorial Policy Checklist.

Statistics

For all statistical analyses, confirm that the following items are present in the figure legend, table legend, main text, or Methods section.

n/a	Confirmed	
☐	☒	The exact sample size (*n*) for each experimental group/condition, given as a discrete number and unit of measurement
☒	☐	A statement on whether measurements were taken from distinct samples or whether the same sample was measured repeatedly
☐	☒	The statistical test(s) used AND whether they are one- or two-sided<br>*Only common tests should be described solely by name; describe more complex techniques in the Methods section.*
☒	☐	A description of all covariates tested
☒	☐	A description of any assumptions or corrections, such as tests of normality and adjustment for multiple comparisons
☐	☒	A full description of the statistical parameters including central tendency (e.g. means) or other basic estimates (e.g. regression coefficient) AND variation (e.g. standard deviation) or associated estimates of uncertainty (e.g. confidence intervals)
☐	☒	For null hypothesis testing, the test statistic (e.g. *F*, *t*, *r*) with confidence intervals, effect sizes, degrees of freedom and *P* value noted<br>*Give P values as exact values whenever suitable.*
☒	☐	For Bayesian analysis, information on the choice of priors and Markov chain Monte Carlo settings
☒	☐	For hierarchical and complex designs, identification of the appropriate level for tests and full reporting of outcomes
☒	☐	Estimates of effect sizes (e.g. Cohen's *d*, Pearson's *r*), indicating how they were calculated

Our web collection on statistics for biologists contains articles on many of the points above.

Software and code

Policy information about availability of computer code

Data collection	AxioVision software (v 4.7)<br>Olympus FV31S-SW (v 2.6.1.243)<br>BD FACSDiva Software (v6.1.3)
Data analysis	All data was plotted and analysed in GraphPad Prism (v 10.4.1). 3D Timelapse confocal microscopy images were analysed using Imaris (v7.7.2). All other images were analysed using FIJI (v 2.9.0). Structures were predicted using AlphaFold3 and analysed in PyMOL (v 2.6.0a0). Phylogenetic analysis was carried out in MEGA (v 11.0.13).

For manuscripts utilizing custom algorithms or software that are central to the research but not yet described in published literature, software must be made available to editors and reviewers. We strongly encourage code deposition in a community repository (e.g. GitHub). See the Nature Portfolio guidelines for submitting code & software for further information.

Data

Policy information about availability of data

All manuscripts must include a data availability statement. This statement should provide the following information, where applicable:

- Accession codes, unique identifiers, or web links for publicly available datasets
- A description of any restrictions on data availability
- For clinical datasets or third party data, please ensure that the statement adheres to our policy

The Plasmodium falciparum protein database used in this study can be accessed at PlasmoDB, release 67. The mass spectrometry proteomics data have been

deposited to the ProteomeXchange Consortium via the PRIDE partner repository with the dataset identifier PXD066159. AlphaFold3 predicted structures and code for FFAT analysis have been deposited in the Zenodo public repository. All other data are included in the article and supplementary data files. Source data are provided.

Research involving human participants, their data, or biological material

Policy information about studies with human participants or human data. See also policy information about sex, gender (identity/presentation), and sexual orientation and race, ethnicity and racism.

Reporting on sex and gender	No research involving human participants, or their data; Human red blood cells were purchased as a medicinal product (transfusion blood, EKSF SAG-M, approval 10569a/96-1) and neither gender or sex of the donors was known to us.
Reporting on race, ethnicity, or other socially relevant groupings	No research involving human participants, or their data; Human red blood cells were purchased as a medicinal product (transfusion blood, EKSF SAG-M, approval 10569a/96-1), and race, ethnicity, or socially relevant groupings of the donors were not known to us.
Population characteristics	No research involving human participants, their data, or biological material.
Recruitment	No research involving human participants, their data, or biological material.
Ethics oversight	No research involving human participants, their data, or biological material.

Note that full information on the approval of the study protocol must also be provided in the manuscript.

Field-specific reporting

Please select the one below that is the best fit for your research. If you are not sure, read the appropriate sections before making your selection.

[x] Life sciences [ ] Behavioural & social sciences [ ] Ecological, evolutionary & environmental sciences

For a reference copy of the document with all sections, see nature.com/documents/nr-reporting-summary-flat.pdf

Life sciences study design

All studies must disclose on these points even when the disclosure is negative.

Sample size	Sample size was not predetermined by any statistical method. For qualitative microscopy experiments, at least 10 images per sample/replicate were collected across 3 replicates for epifluorescence microscopy, and across 2 replicates for confocal and ultrastructure expansion microscopy. For microscopy images used for quantifications, at least 20 images were acquired across 3 replicates, with the exception of the images for the PH binding to the PPM in Fig. 6h and Supplementary Fig. 10i, in which cases only 10 and 14 images across 3 replicates were considered for the ones co-expressing the HEPN-L domain, and only 5 images across 1 replicate were considered for the ones expressing the PH domain alone, as repeated qualitatitave analysis using epifluorescence microscopy had already determined a cytosolic signal so no further replicates were added after confirmation of such a signal in randomly chosen cells. In most experiments all images were considered for the analysis, with the exception of the IMC perimeter measurements which were taken from 15-19 cells per sample/replicate chosen randomly from the total of cells.
Data exclusions	No data was excluded.
Replication	At least three biological replicates on different days were peformed for most experiments. Selected experiments were performed in less than three replicates as indicated in the respective legends.
Randomization	Imaged cells were chosen randomly during experiments.
Blinding	When comparing control and experimental groups on microscopy experiments related to PfVPS13L1 investigators were blinded in the initial replicate. However, given striking differences in phenotype and growth, blinding was omitted in the following replicates as control and experimental groups are immediately distinguishable.

Reporting for specific materials, systems and methods

We require information from authors about some types of materials, experimental systems and methods used in many studies. Here, indicate whether each material, system or method listed is relevant to your study. If you are not sure if a list item applies to your research, read the appropriate section before selecting a response.

Materials & experimental systems

n/a	Involved in the study
	☒ Antibodies
☒	☐ Eukaryotic cell lines
☒	☐ Palaeontology and archaeology
☒	☐ Animals and other organisms
	☒ Clinical data
☒	☐ Dual use research of concern
☒	☐ Plants

Methods

n/a	Involved in the study
☒	☐ ChIP-seq
	☒ Flow cytometry
☒	☐ MRI-based neuroimaging

Antibodies

Antibodies used

Used for staining in Ultra-expansion microscopy (U-ExM):
As primary antibodies: mouse anti-V5 (1/250 dilution, BioRad #MCA1360, clone SV5-Pk1, lot 162949), mouse anti-αTubulin (1/500 dilution, Thermo Fischer #32-3500, clone B-5-1-2, lot WJ337893), rabbit anti-mCh (1/1000 dilution, abcam #ab167453, clone EPR20579, lot 1061130-4), rabbit anti-MORN1 (1/1000 dilution, kind gift by B. Striepen).
As secondary antibodies: anti-mouse IgG Alexa Fluor™ 594 (Thermo Fischer #A11032, lot 2301112), anti-mouse IgG Alexa Fluor™ 633 (Thermo Fischer #A21053, lot 948495), anti-rabbit IgG Alexa Fluor™ 546 (Thermo Fischer #A10040, lot 948483) and anti-rabbit IgG Alexa Fluor™ 647 (Thermo Fischer #A21244, lot 1386544), all used 1/500)

Validation

Three of the primary antibodies used in this study are standard commercial antibodies, validated by the manufacturer and previously used in other studies for this specific application (U-ExM).
- mouse anti-V5 (1/250 dilution, BioRad #MCA1360, clone SV5-Pk1): Validated by manufacturer for immunofluorescence assays. Used for UExM in PMIDs: 36993606, 37400439, 38829893.
- mouse anti-αTubulin (1/500 dilution, Thermo Fischer #32-3500, clone B-5-1-2): Validated by manufacturer for immunofluorescence assays. Used for UExM in PMIDs: 36993606
- rabbit anti-mCh (1/1000 dilution, abcam #ab167453): Validated by manufacturer for immunofluorescence assays. Used for UExM in PMIDs: 36524422
The other antibody, anti-MORN1, was generated and validated in a previous study. (PMIDs: 15525431)

Clinical data

Policy information about clinical studies
All manuscripts should comply with the ICMJE guidelines for publication of clinical research and a completed CONSORT checklist must be included with all submissions.

Clinical trial registration

Human red blood cells were purchased from the university clinic Hamburg Eppendorf (UKE) which sells this as a medicinal product. The UKE has approval to sell this product (transfusion blood, EKSF SAG-M) from the Paul-Ehrlich-Institute (German authority to approve pharmaceutical products, approval 10569a/96-1). No information on donors was known to us.

Study protocol

NA

Data collection

NA

Outcomes

NA

Plants

Seed stocks

Report on the source of all seed stocks or other plant material used. If applicable, state the seed stock centre and catalogue number. If plant specimens were collected from the field, describe the collection location, date and sampling procedures.

Novel plant genotypes

Describe the methods by which all novel plant genotypes were produced. This includes those generated by transgenic approaches, gene editing, chemical/radiation-based mutagenesis and hybridization. For transgenic lines, describe the transformation method, the number of independent lines analyzed and the generation upon which experiments were performed. For gene-edited lines, describe the editor used, the endogenous sequence targeted for editing, the targeting guide RNA sequence (if applicable) and how the editor was applied.

Authentication

Describe any authentication procedures for each seed stock used or novel genotype generated. Describe any experiments used to assess the effect of a mutation and, where applicable, how potential secondary effects (e.g. second site T-DNA insertions, mosiacism, off-target gene editing) were examined.

Flow Cytometry

Plots

Confirm that:

- [ ] The axis labels state the marker and fluorochrome used (e.g. CD4-FITC).
- [ ] The axis scales are clearly visible. Include numbers along axes only for bottom left plot of group (a 'group' is an analysis of identical markers).
- [ ] All plots are contour plots with outliers or pseudocolor plots.
- [ ] A numerical value for number of cells or percentage (with statistics) is provided.

Methodology

Sample preparation	Samples were stained with Hoechst 33342 and Dihydroethidium for 20 minutes at room temperature, and the parasites were inactivated and the staining stopped with 400 µl of 0.003% glutaraldehyde (Roth #4157) containing medium.
Instrument	BD LSRII flow cytometer
Software	BD FACSDiva Software (v6.1.3)
Cell population abundance	*Describe the abundance of the relevant cell populations within post-sort fractions, providing details on the purity of the samples and how it was determined.*
Gating strategy	Flow cytometry was merely used to quantify parasitemia (% of infected RBCs over total), which is why no plots are included in our manuscript. Gating strategies were standard ones used in many publicatoins to differentiate between Hoechst and DHE stained cells vs unstained. An example of the gating strategy can be found in Fig S8F of PMID: 31568532.

- [ ] Tick this box to confirm that a figure exemplifying the gating strategy is provided in the Supplementary Information.
